# Differences in female athlete triad risk factors between Japanese and American female runners: A comparative study

**DOI:** 10.1002/pmrj.70099

**Published:** 2026-02-11

**Authors:** Ana Carla C. Salamunes, Natsue Koikawa, Nancy I. Williams, Kristen J. Koltun, Maha L. Vijaya Krishnan, Yuko Sakurama, Takao Matsuda, Shihoko Suzuki, Yuji Takazawa, Mary Jane De Souza

**Affiliations:** ^1^ Women's Health and Exercise Lab Pennsylvania State University University Park Pennsylvania USA; ^2^ Japanese Center for Research on Women in Sport Juntendo University Tokyo Japan; ^3^ Graduate School of Health and Sports Science Juntendo University Chiba Japan; ^4^ Neuromuscular Research Laboratory, Department of Sports Medicine and Nutrition University of Pittsburgh Pittsburgh Pennsylvania USA; ^5^ College of Medicine Drexel University Philadelphia Pennsylvania USA; ^6^ Department of Gynecology, National Hospital Organization Nishi‐Beppu National Hospital Oita Japan; ^7^ Faculty of Health and Social Services Kanagawa University of Human Services Kanagawa Japan; ^8^ Department of Orthopaedics Surgery, Faculty of Medicine Juntendo University Tokyo Japan

## Abstract

**Background:**

Differences in female athlete triad risk factors between runners from Japan and the United States have not been explored.

**Objective:**

To compare the prevalence of Triad risk factors and Triad components between female Japanese and American distance runners.

**Design:**

Observational, cross‐sectional.

**Setting:**

Two universities; one in the United States and one in Japan.

**Participants:**

A total of 77 female runners: 36 Japanese middle‐ and long‐distance runners, and 41 American distance runners.

**Interventions:**

Not applicable.

**Main Outcome Measures:**

Triad components were energy deficiency, menstrual disturbances, and poor bone health. Triad risk factors were, respectively for each component: body mass index, serum total triiodothyronine, and history of diagnosed eating disorders; delayed menarche, history of menstrual irregularity, and current oligoamenorrhea; and bone mineral density and history of stress fractures. Prevalence was compared with chi‐square and Fisher's exact tests. Group differences were assessed with *t*‐tests and Mann–Whitney *U* tests. Data are mean difference (95% confidence interval [CI]).

**Results:**

Japanese runners had lower body weight (−4.15 [95% CI, −7.03.to −1.27] kg, *p* = .005) and percentage of body fat (−5.2 [95% CI, −6.9 to −3.5] %, *p* < .001) than American runners, with no differences in body mass index, serum total triiodothyronine, age of menarche, or menstrual cycle length (*p* > .05). Japanese runners had significantly lower bone mineral density at the lumbar spine (−0.195 [95% CI, −0.246 to −0.143] g/cm^2^, *p* < .001), total hip (−0.115 [95% CI, −0.165 to −0.064] g/cm^2^, *p* < .001), and femoral neck (−0.214 [95% CI, −0.260 to −0.167] g/cm^2^, *p* < .001), but higher total hip *Z*‐scores than the American runners (0.5 [95% CI, 0.1 to 1.0], *p* = .022). The prevalence of energy deficiency (56% vs. 32%, *p* = .035), history of stress fractures (50% vs. 17%, *p* = .003), and of runners experiencing all three Triad components (42% vs. 16%, *p* = .014) was higher in Japanese than American runners. No other differences in prevalence were observed.

**Conclusions:**

Japanese female runners may be at a higher Triad risk than American peers, given their higher prevalence of energy deficiency risk factors, history of stress fractures, and of athletes experiencing all Triad components.

## INTRODUCTION

The female athlete triad (Triad)—a common condition among female athletes participating in leanness and endurance sports—is caused by insufficient energy intake relative to energy expenditure and consists of three interrelated components: energy deficiency with or without disordered eating, menstrual dysfunction, and poor bone health.^1^, ^2^ Each Triad component may be assessed from multiple risk factors, such as low total triiodothyronine (TT_3_) and evidence of disordered eating behavior, irregular or absent menstrual cycles, and low bone mineral density (BMD) *Z*‐scores and the incidence of stress fractures, respectively.^1^ All of these Triad risk factors are also described as primary indicators of the relative energy deficiency in sport (REDs) model.^3^ Even though the prevalence of the Triad conditions has been studied in countries of several continents, i.e., America, Europe, Africa, Asia, and Oceania,^4^, ^5^, ^6^, ^7^, ^8^, ^9^, ^10^, ^11^, ^12^, ^13^, ^14^, ^15^, ^16^, ^17^, ^18^, ^19^ studies exploring racial and ethnic difference in Triad risk factors and components are still warranted. Understanding differences in the prevalence of Triad risk factors across multiple racial and ethnic groups is critical for providing individualized care, implementing prevention, early intervention, and monitoring strategies, developing culturally sensitive approaches, and providing race‐ and ethnicity‐specific recommendations to be adopted by psychologists, nutritionists, sports dietitians, and other health care professionals.

Notable cultural and physiological differences between American and Japanese individuals have been reported, including dietary habits,^20^, ^21^, ^22^ body image,^23^ and bone health.^24^, ^25^ However, few studies compared Triad‐related conditions between athletic groups from the two populations. An investigation demonstrated that Japanese female track athletes trained for longer hours, slept less, and scored higher on the Eating Attitudes Test‐26 compared to American female track athletes.^23^ These findings might indicate that Japanese athletes have less recovery time and are more likely to experience disordered eating/eating disorders than American athletes and therefore may have a higher risk for energy deficiency. Moreover, premenopausal Asian‐American—from Chinese, Japanese, Philippine, Vietnamese, and Korean origin—women living in the United States were reported to have lower BMD than white women from the same country, an outcome that was associated with differences in body size.^26^, ^27^ If Japanese athletes are more susceptible to disordered eating behavior and/or eating disorders and lower BMD, an exposure to energy deficiency in that population may impose an even greater risk for bone stress injury, including stress fractures, compared to American athletes given their smaller bone size.

The aims of this study were (1) to compare the prevalence of Triad risk factors and of the three Triad components in female Japanese and American distance runners, and (2) to compare Triad‐related health indicators in female Japanese and American distance runners, including energy intake (EI), energy availability (EA), serum TT_3_, insulin‐like growth factor‐1 (IGF‐1), body weight (BW), body mass index (BMI), body composition, menstrual status, and BMD. We hypothesized that the prevalence of energy deficiency and poor bone health would be higher in Japanese runners than American runners. We also hypothesized that Japanese runners would weigh less and have a lower percentage of body fat, serum TT_3_, and BMD than American runners, despite similar EI and a similar prevalence of menstrual disturbances.

## METHODS

### Study design

The present study is an observational, comparative cross‐sectional analysis of a dataset derived from two studies: (1) a cross‐sectional study that originally tested Japanese female collegiate and club team athletes and para‐athletes (unpublished), from which only data from middle‐ and long‐distance runners (not para‐athletes) were used; and (2) a longitudinal study on American female Division I and university‐affiliated club team long‐distance runners tested during pre‐ and off‐season,^28^, ^29^ from which only the preseason data were used. Studies were approved by the institutional review boards of each university; in Japan and in the United States. Participants signed written informed consent forms prior to participation. The present study compared the BW, BMI, body composition, BMD, exercise time, exercise energy expenditure (EEE), EI, EA, macronutrient intake, metabolic hormones, and self‐reported history of Triad risk factors (menstrual disturbances, diagnosed eating disorders, stress fractures) between American and Japanese runners. The prevalence of each Triad risk factor, and of individual and combined Triad components was assessed and compared between Japanese and American runners. The tested Triad components and risk factors are detailed in Table 1. Even though more indicators are suggested to be indicative of energy deficiency in the REDs Consensus Statement,^3^ all primary indicators of REDs for adult females (ie, amenorrhea, low TT_3_, high‐risk bone stress injury, low BMD, and eating disorders/disordered eating) and two of the four REDs secondary indicators (oligomenorrhea and low‐risk bone stress injury) are also Triad risk factors. The remaining two REDs secondary indicators (high low‐density lipoprotein cholesterol and depression and/or anxiety) and several REDs “potential indicators” still lack robust evidence demonstrating they are caused by energy deficiency.^3^ Therefore, for the purpose of this comparative analysis, it was most appropriate to include only the risk factors that have robust evidence demonstrating association with energy deficiency, the risk factors already assessed in the Triad model.

**TABLE 1:** Female athlete Triad components and the respective Triad risk factors that were compared between Japanese and American female middle‐ and long‐distance runners.

Triad component	Energy deficiency	Menstrual disturbances	Poor bone health
Triad risk factors	BMI <18.5 kg/m^2^ Self‐reported history of diagnosed eating disorders TT_3_ < 80 ng/dL (<1.232 nmol/L)	Delayed menarche (age of menarche ≥14 years) Self‐reported history of menstrual irregularities Current oligoamenorrhea^a^	Self‐reported history of stress fractures Low BMD (*Z*‐score <−1.0 at any site^b^)

Abbreviation: BMD, bone mineral density; BMI, body mass index; TT_3_, total triiodothyronine.

^a^ Oligomenorrhea was defined as menstrual cycles lasting 36–90 days. Amenorrhea was defined as menstrual cycles lasting >90 days.

^b^ BMD sites were: total body, lumbar spine (L_1_‐L_4_), total hip, and femoral neck.

### Participants

Participants were female runners ages 17–25 years from Japan and from the United States. The Japanese cohort included 36 athletes (9 middle‐distance, 25 long‐distance, and 2 middle‐ and long‐distance runners). The U.S. cohort included  41 collegiate distance runners (24 from the Division I cross‐country varsity team and 17 from a university‐affiliated club team). All participants reported having been assigned to female biological sex at birth, and all identified as women (gender). Inclusion criteria for the study in American distance runners were reporting good health; actively participating in competitive and/or club/recreational‐level distance running on a team or as an individual; actively training for a running event of at least 5 km in length; and having been cleared by the sports medicine physician (for Division I varsity runners) or cleared as “low risk” using the Risk Stratification from the American College of Sports Medicine Guidelines for Exercise Testing and Prescription^30^ (if member of a university‐affiliated club team). Exclusion criteria were smoking; pregnancy or lactation; ineligible to be a varsity athlete (ie, injured, academically ineligible) and/or ineligible to participate in regular distance running (eg, injured); and identified as “moderate” or “high risk” using the American College of Sports Medicine Risk Stratification Guidelines.^30^

The study in Japan (*n* = 61) included female athletes and para‐athletes competing in track and field, swimming, beach volleyball, and goalball, aged 18–34 years old. In this analysis, only middle‐ and long‐distance runners were included. Runners were from a Japanese university's track and field club (*n* = 23) or club sports teams (*n* = 15) aged 18–30 years. Japanese para‐athletes (*n* = 3), athletes who were not middle‐ or long‐distance runners (*n* = 20), and runners >25 years (*n* = 2) were excluded from this analysis, therefore this analysis included *n* = 36 Japanese runners and *n* = 41 American runners. Inclusion criteria for the study in Japanese athletes were: reporting good health; actively participating in competitive running, training for at least 2 hours/day, 5 days/week; actively training for a running event of at least 5 km in length (for long‐distance runners) or for a running event of 800–3000 m in length (for middle‐distance runners). Exclusion criteria were: smoking, pregnancy or lactation, and ineligibility to participate in normal training activities (eg, injury).

### Anthropometrics and body composition

Height was measured to the nearest 0.1 cm with a stadiometer (United States) or a digital stadiometer (A&B Co., Ltd) (Japan). BW, body composition (total fat mass [FM], lean body mass, fat‐free mass [FFM], and percentage of body fat) and BMD (total body BMD, total body *Z*‐score, lumbar spine (L_1_‐L_4_) BMD, L_1_‐L_4_ *Z*‐score, total hip BMD, total hip *Z*‐score, femoral neck BMD, and femoral neck *Z*‐score) were assessed with dual‐energy ray absorptiometry (DXA) (United States: GE Lunar iDXA, GE Healthcare, Madison, WI, USA; Japan: Hologic Discovery A, X‐Ray Bone Densitometer, Hologic, Bedford, MA). BMI was calculated (kg/m^2^). DXA scans on American distance runners were performed by an International Society for Clinical Densitometry‐certified technician. DXA scans on Japanese distance runners were performed by an orthopedic physician affiliated with the university hospital in Japan. One American runner did not complete the DXA scans.

At the time of study data collection, the DXA scanner software used in American runners did not include a pediatric database for hip scans; therefore, *Z*‐scores for the total hip and femoral neck sites were not available for American runners age < 20 years (*n* = 22). Additionally, total body and lumbar spine *Z*‐scores were not available for two American runners because they did not identify with any of the ethnic groups listed in the DXA scanner software database. Therefore, the missing *Z*‐scores were calculated using the revised reference curves from the Bone Mineral Density in Childhood Study.^31^ Because the reference curves were designed for Hologic DXA scanners but the American runners were tested with a GE Lunar DXA scanner, a cross‐calibration equation was used to recalculate the missing total hip, femoral neck, and lumbar spine BMD values to match those of a Hologic scanner. The recalculated BMD values were then used to calculate the *Z*‐scores following the Bone Mineral Density in Childhood Study.^31^

### Health history

A survey including health history, race/ethnicity exercise, and nutrition information was used to assess self‐reported history of diagnosed eating disorders, menstrual status and history, and history of stress fractures. Racial/ethnic categories listed herein reflect the revised United States 2024 Statistical Policy Directive No. 15, which assesses multiple races (physical traits) and ethnicities (cultural aspects) in a single question.^32^ Participants who did not identify with any of the options were categorized as “other.” Menstrual‐related questions included age of menarche, average menstrual cycle length, history of menstrual irregularity, and current menstrual status, that is, currently experiencing regular cycles, irregular cycles, or hormonal contraceptive use. History of menstrual irregularity was reported in response to a yes/no question asking if the participant had ever gone through any length of time without menstruating regularly. Participants were classified as eumenorrheic if current menstrual cycle length was <36 days, as oligomenorrheic if cycles were 36–90 days, and as amenorrheic if current menstrual cycle length was >90 days. Participants who reported current hormonal contraceptive use (oral contraceptives: *n* = 2 Japanese, *n* = 10 American) were excluded from the current menstrual status and menstrual cycle length analyses. Gynecological age was calculated as age – age of menarche (years). Six American runners did not complete the Health, Exercise, and Nutrition Survey, of whom age of menarche was collected from two participants. Two American runners reported not knowing if they had experienced stress fractures, therefore they were excluded from the stress fractures analysis.

### Energy intake

Participants received written and oral instructions to complete food logs for 3 consecutive days (2 weekdays, 1 weekend day). Assessing EI for 3 days induces low participant burden in order to improve participant compliance.^33^ Participants were instructed to record any foods and beverages consumed, as precisely as possible. Portion sizes were demonstrated to assist in accuracy. For American runners, coded data were interpreted with Nutritionist Pro (Version 3.1, Axxya Systems, Stafford, TX). For the Japanese runners, food records were verified by a certified sports dietitian and interpreted with Nutrition Plus (Kenpakusha Co., Ltd., Tokyo) based on the Japanese Standard Tables of Food Composition.^34^ EI was calculated as kcal/day and relative to BW (EI/BW) (kcal/kg BW/day), and macronutrient intake data was reported in g/day and relative to BW (g/kg BW/day). Dietary data were missing for  14 American runners.

### Exercise energy expenditure and energy availability

Participants self‐reported exercise activity descriptions, durations, and dates in 7‐day exercise logs. The 2011 Compendium of Physical Activities was used to estimate each activity's metabolic equivalent of task (METs),^35^ with one MET representing the energy cost of resting. The amount of METs used to calculate the EEE for each activity were the activity METs minus 1 (adjusted METs), to avoid including the resting energy expenditure as part of EEE. The total EEE (kcal/week) and total exercise time (min/week) are reported herein. EA was calculated as EI‐EEE/kg FFM/day (kcal/kg FFM/day). Data from the exercise logs were unclear for 15 Japanese runners, and were therefore not used. Exercise data were missing from 17 American runners.

### Serum total triiodothyronine and insulin‐like growth factor 1

Blood samples were obtained from the antecubital vein, in the morning following a 12‐hour fast using blood collection tubes (BD Vacutainer; Becton, Dickinson and Company, Franklin Lakes, NJ, for American runners). Samples were allowed to clot at room temperature for a minimum of 30 minutes before being centrifuged for 15 minutes at 4°C (Eppendorf centrifuge 5804R; Eppendorf, Hamburg, Germany), aliquoted, and stored at −80°C for future analysis. Serum hormone concentrations were measured in American runners with competitive immunoassays using chemiluminescence immunoassay analyzers (IGF‐1: IDS‐iSYS; Immunodiagnostic Systems Limited, Gaithersburg, MD; TT_3_: Immulite, Diagnostic Products Corporation, Los Angeles, CA). Analytical sensitivity of the assays were 8.8 ng/mL and 35 ng/dL, respectively for IGF‐1 and TT_3_. In Japanese runners, serum hormone levels were measured at SRL Inc. (Tokyo, Japan) with chemiluminescent enzyme immunoassays for TT_3_ and with electrochemiluminescence immunoassays for IGF‐1. IGF‐1 data were available only for 17 American runners, and TT_3_ data were available for 36 American runners. No differences in TT_3_ and IGF‐1 were observed between runners taking and not taking oral contraceptives (*p* > .05); therefore, runners taking oral contraceptives were not excluded from these analyses.

Serum TT_3_ is frequently used as a marker of energy deficiency, following demonstrations that it is affected by short‐term^36^, ^37^, ^38^, ^39^, ^40^ and long‐term^41^ inductions of energy deficiency and its low concentrations in females with amenorrhea.^42^, ^43^ Notably, TT_3_ is suggested to be used as a marker of energy deficiency in both Triad^1^ and REDs models.^3^ Therefore, low TT_3_ (<80 ng/dL or <1.232 nmol/L) was considered a risk factor for the energy deficiency component of the Triad.

### Data analysis

#### Triad risk factors and Triad components

The prevalence of current and/or previous Triad risk factors and Triad components was assessed separately for Japanese and American runners. Triad components were energy deficiency, menstrual disturbances, and poor bone health. Triad risk factors were symptoms, conditions, or measurements suggesting the presence of a Triad component. The Triad components and respective risk factors that were included in this study are detailed in Table 1.

The following are reported for each group:

1. Prevalence of each Triad risk factor: low BMI, low TT_3_, history of diagnosed eating disorders, history of menstrual irregularity, current oligo‐amenorrhea, delayed menarche, low BMD, and history of stress fractures;
2. Prevalence of ≥1 energy deficiency risk factor: the percentage of participants with low BMI and/or low TT_3_ and/or history of diagnosed eating disorders;
3. Prevalence of ≥1 menstrual disturbance risk factor: the percentage of participants with history of menstrual irregularity and/or current oligoamenorrhea and/or delayed menarche;
4. Prevalence of ≥1 poor bone health risk factor: the percentage of participants with low BMD and/or history of stress fractures;
5. Prevalence of ≥1 Triad component: the percentage of participants with ≥1 Triad risk factor from any of the three Triad components;
6. Prevalence of ≥2 Triad components: the percentage of participants with ≥2 Triad risk factors from a minimum of two Triad components (eg, ≥1 energy deficiency risk factor and ≥1 menstrual disturbance risk factor);
7. Prevalence of all three Triad components: the percentage of participants with ≥1 energy deficiency risk factor and ≥1 menstrual disturbance risk factor and ≥1 poor bone health risk factor.

Because of missing datapoints, only participants who had completed the assessments of at least one Triad risk factor for each Triad component (*n* = 36 Japanese, *n* = 38 American) were included in the latter analysis (ie, items 5–7 of the list).

### Statistical analysis

Normal distribution was assessed for numeric variables with the Shapiro–Wilk test and qualitatively from histograms and quantile‐quantile plots. Variables that were not normally distributed were analyzed with non‐parametric tests (ie, age, age of menarche, menstrual cycle length, FM, EI [kcal/day], carbohydrate intake [g/day], protein intake [g/kg BW/day], EEE, TT_3_, and total body *Z*‐score). Numeric variables were compared between American and Japanese runners with either independent samples *t*‐tests or Mann–Whitney *U* tests. For *t*‐tests, equal variances were assumed for nonsignificant Levene's test results (*p* > .05), and unequal variances were assumed otherwise. Chi‐square tests (or Fisher's exact tests, for groups with less than five observations for a given condition) were conducted to compare the proportions of Triad risk factors and Triad components between Japanese and American runners. Data are shown as mean ± SD, or as median (interquartile range) if non‐normally distributed. Group differences are described as mean/median (95% confidence interval [CI]). Significance level was *p* < .05.

## RESULTS

**FIGURE 1:**
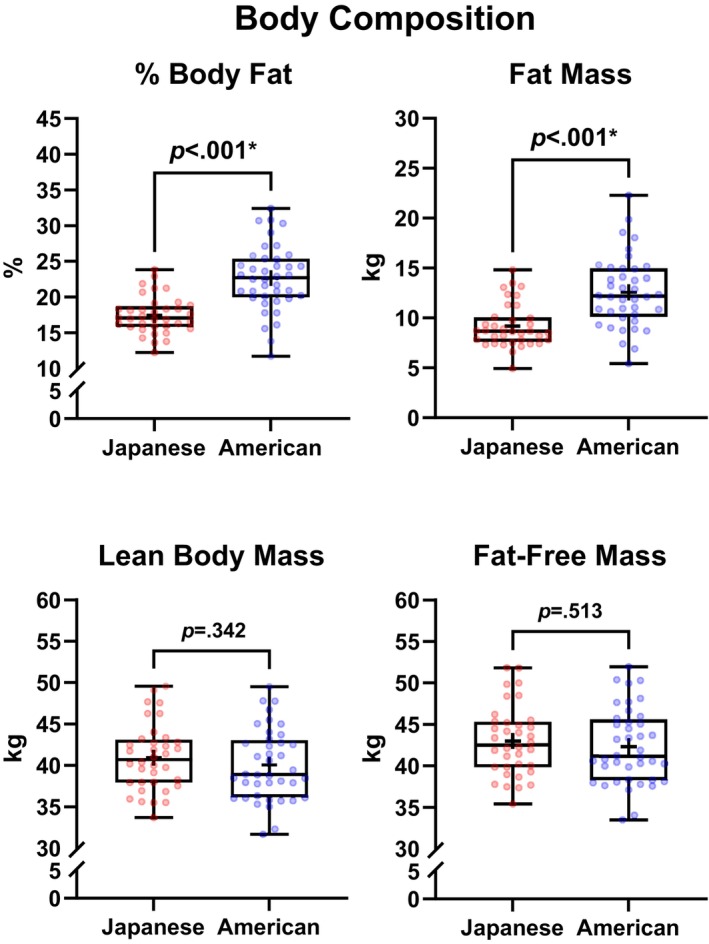
Differences in body composition between Japanese and American female distance runners assessed with dual‐energy x‐ray absorptiometry. Box and whisker plots represent the minimum, 25th percentile, median, 75th percentile, and maximum values, and (+) signs represent the means for each group. *Statistically significant group differences (*p* < .05).

Table 2 presents the demographics and group comparisons between Japanese and American female runners. No statistically significant group differences were observed in age of menarche (*p* = .149), gynecological age (*p* = .428), or menstrual cycle length (*p* = .125). Compared to American runners, Japanese runners were significantly older (*p* = .043), shorter (*p* = .014), and weighed less (*p* = .005), but had similar BMI (*p* = .080). As depicted in Figure 1, Japanese runners had significantly lower percentage of body fat compared to American runners (17.5 ± 2.6 vs. 22.7 ± 4.6%; −5.2 [95% CI, −6.9 to −3.5] %, *p* < .001), which was explained by statistical differences in FM (8.7 [2.5] vs. 12.2 [4.8] kg; −3.3 [95% CI, −4.6 to −1.9] kg, *p* < .001), but not in lean body mass (41.0 ± 4.0 vs. 40.0 ± 4.3 kg; +0.9 [95% CI, −1.0 to 2.8] kg, *p* = .342) or FFM (43.0 ± 4.2 vs. 42.3 ± 4.5 kg; +0.7 [95% CI, −1.3 to 2.7] kg, *p* = .513).

**TABLE 2:** Demographics and group differences between Japanese and American middle‐ and long‐distance runners. Data are mean ± SD or median (IQR).

	Japanese	American	Difference [95% CI]	*p* value
Age, (y)	20 (2)	19 (2)	1.0 [0.0 to 1.0]	.043*
Height (cm)	161.7 ± 4.6	164.8 ± 6.1	−3.14 [−5.62 to −0.66]	.014*
Weight (kg)	51.0 ± 5.8	55.2 ± 6.7	−4.15 [−7.03 to −1.27]	.005*
Body mass index (kg/m^2^)	19.5 ± 1.8	20.3 ± 2.2	−0.83 [−1.75 to 0.10]	.080
Age of menarche, (y)	14 (2)	13 (1)^b^	1.0 [0.0 to 1.0]	.149
Gynecological age (y)	6.3 ± 1.9	5.9 ± 1.9^b^	0.36 [−0.54 to 1.26]	.428
Menstrual cycle length (days)	28 (6)^c^	30 (7)^d^	−3.0 [−6.0 to 1.0]	.125
Race/ethnicity (% (*n*))^a^				
Asian	100 (36)	0 (0)	‐	‐
White	0 (0)	90.0 (36)	‐	‐
Black or African American	0 (0)	2.5 (1)	‐	‐
Hispanic or Latino	0 (0)	2.5 (1)	‐	‐
Other	0 (0)	5.0 (2)	‐	‐

*Note*: Gynecological age was calculated as age – age of menarche (years).

Abbreviations: CI, confidence interval; IQR, interquartile range.

^a^ American runners *n* = 40.

^b^ American runners *n* = 37.

^c^ Japanese runners *n* = 28.

^d^ American runners *n* = 22.

* Significant differences between Japanese and American runners.

Table 3 shows group differences in diet, exercise, and hormone concentrations. Even though no statistically significant difference in EI was observed between groups (*p* = .261), EI/BW was higher in Japanese runners than American runners (*p* = .023). Japanese runners reported consuming more carbohydrates relative to BW than American runners (*p* = .002), with no differences in protein (*p* = .098) and fat intake (*p* = .879). Compared to American runners, Japanese runners reported higher exercise volume (*p* < .001) and EEE (*p* = .017), but similar EA (*p* = .343); however, the sample size was notably smaller for the exercise data analyses, due to missing data points. Even though IGF‐1 was significantly lower in Japanese runners compared to American runners (*p* = .020), TT_3_ concentrations were not significantly different between groups (*p* = .752).

**TABLE 3:** Differences in energy intake, macronutrient intake, exercise habits, and energetic hormone concentrations between Japanese and American middle‐ and long‐distance runners. Data are mean ± SD or median (IQR).

	Japanese	American	Difference [95% CI]	*p* value
Diet^a^				
Energy intake (kcal/d)	2094 (306)	2071 (567)	103 [−76 to 355]	.261
Energy intake (kcal/kg BW/d)	43.1 ± 8.3	37.6 ± 10.4	5.50 [0.79 to 10.22]	.023*
Carbohydrates (g/d)	294 (54)	270 (80)	26.31 [−5.66. 60.44]	.098
Carbohydrates (g/kg BW/d)	5.97 ± 1.27	4.93 ± 1.27	1.04 [0.39 to 1.69]	.002*
Fats (g/d)	66.0 ± 13.1	72.3 ± 27.2	−6.33 [−17.83 to 5.18]	.272
Fats (g/kg BW/d)	1.32 ± 0.33	1.33 ± 0.52	−0.02 [−0.23 to 0.20]	.879
Proteins (g/d)	84.9 ± 14.7	86.3 ± 30.5	−1.44 [−14.35 to 11.46]	.822
Proteins (g/kg BW/d)	1.73 (0.44)	1.56 (0.59)	0.19 [−0.05 to 0.42]	.098
Exercise				
Exercise time (min/wk)	595 ± 203^b^	315 ± 161^c^	280 [170 to 390]	<.001*
EEE (kcal/wk)	4656 (3147)^b^	3242 (3232)^c^	1566 [270 to 2713]	.017*
Energy availability (kcal/kg FFM/d)	32.3 ± 13.2^b^	36.4 ± 14.9^d^	−4.08 [−12.68 to 4.51]	.343
Hormones				
IGF‐1 (nmol/L)	29.1 ± 8.0	34.9 ± 8.7^e^	−5.8 [−10.6 to −0.9]	.020*
TT_3_ (nmol/L)	1.41 (0.40)	1.48 (0.35)^f^	−0.03 [−0.15 to 0.11]	.752

Abbreviations: BW, body weight; CI, confidence interval; EEE, exercise energy expenditure; FFM, fat‐free mass; IGF‐1, insulin‐like growth factor‐1; IQR, interquartile range; TT_3_, total triiodothyronine.

^a^ American runners *n* = 27.

^b^ Japanese runners *n* = 21.

^c^ American runners *n* = 24.

^d^ American runners *n* = 23.

^e^ American runners *n* = 17.

^f^ American runners *n* = 36.

* Significant differences between Japanese and American runners.

**FIGURE 2:**
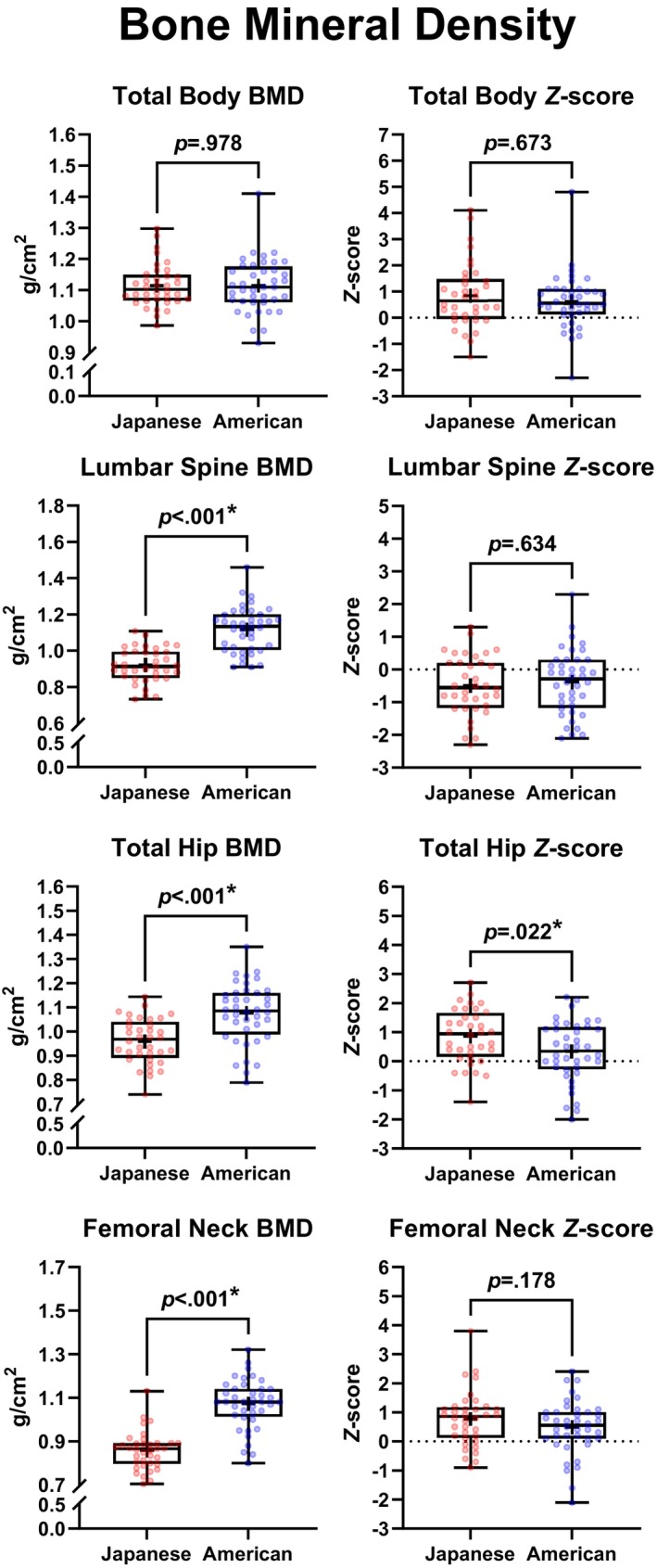
Differences in BMD and BMD *Z*‐scores between Japanese and American female distance runners, assessed with dual‐energy x‐ray absorptiometry. Box and whisker plots represent the minimum, 25th percentile, median, 75th percentile, and maximum values, and (+) signs represent the means for each group. *Statistically significant group differences (*p* < .05). BMD, bone mineral density.

**FIGURE 3:**
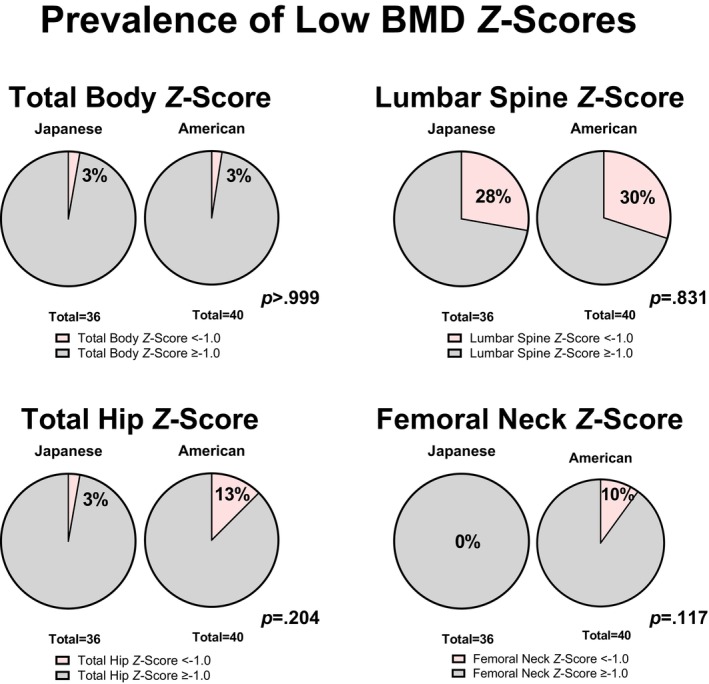
Prevalence of bone mineral density *Z*‐score < −1.0 in Japanese and American female distance runners, assessed with dual‐energy x‐ray absorptiometry. No significant differences in group proportions were observed. BMD, bone mineral density.

Figure 2 depicts group differences in BMD and *Z*‐scores between Japanese and American runners. Japanese runners had significantly lower lumbar spine BMD (0.921 ± 0.095 vs. 1.115 ± 0.125 g/cm^2^; −0.195 [95% CI, −0.246 to −0.143], *p* < .001), total hip BMD (0.960 ± 0.093 vs. 1.075 ± 0.125 g/cm^2^; −0.115 [95% CI, −0.165 to −0.064], *p* < .001), and femoral neck BMD (0.857 ± 0.086 vs. 1.071 ± 0.115 g/cm^2^; −0.214 [95% CI, −0.260 to −0.167], *p* < .001), but similar total body BMD (1.114 ± 0.069 vs. 1.113 ± 0.087; +0.001 [95% CI, −0.036 to 0.037], *p* = .977). Total hip *Z*‐scores were higher in the Japanese runners, compared to the American runners (0.9 ± 0.9 vs. 0.3 ± 1.0; 0.5 [95% CI, 0.1–1.0], *p* = .022), with no statistically significant differences in *Z*‐scores of the total body (0.7 [1.5] vs. 0.6 [1.0]; 0.1 [95% CI, −0.3 to 0.6], *p* = .669), lumbar spine (−0.5 ± 0.9 vs. −0.4 ± 1.0; −0.1 [95% CI, −0.5 to 0.3], *p* = .635), and femoral neck (0.8 ± 1.0 vs. 0.5 ± 1.0; 0.3 [95% CI, −0.1 to 0.7], *p* = .086). Table 4 and Figure 3 show the proportions of participants with low BMD *Z*‐scores in each group. Most of the low *Z*‐scores were observed at the lumbar spine. No statistically significant differences between the proportions of Japanese and American runners who had low BMD at each site were observed (*p* > .05).

**TABLE 4:** Prevalence of Female Athlete Triad risk factors and Triad components in Japanese and American female distance runners. Data are %(*n*/total *n*).

	Japanese	American	*p* value
Energy deficiency^a^	**55% (20/36)**	**32% (13/41)**	**.035** *
Low BMI (<18.5 kg/m^2^)	28% (10/36)	20% (8/41)	.393
History of eating disorders	8% (3/36)	9% (3/35)	>.999
Low TT_3_ (<80 ng/dL)	28% (10/36)	19% (7/36)	.405
Menstrual disturbances^b^	**92% (33/36)**	**82% (31/38)**	**.310**
Delayed menarche (age of menarche ≥14 y)	67% (24/36)	46% (17/37)	.074
History of menstrual irregularity	81% (29/36)	77% (27/35)	.725
Current Oligoamenorrhea	35% (12/34)	36% (9/25)	.955
Poor bone health^c^	**64% (23/36)**	**50% (20/40)**	**.223**
History of stress fractures	50% (18/36)	17% (6/35)	.003*
Low BMD (*Z*‐score < −1.0 at any site)	28% (10/36)	35% (14/40)	.499
Low total body BMD (*Z*‐score < −1.0)	3% (1/36)	3% (1/40)	>.999
Low lumbar spine BMD (*Z*‐score < −1.0)	28% (10/36)	30% (12/40)	.831
Low total hip BMD (*Z*‐score < −1.0)	3% (1/36)	13% (5/40)	.204
Low femoral neck BMD (*Z*‐score < −1.0)	0% (0/36)	10% (4/40)	.117
≥1 Triad component^d^	**94% (34/36)**	**95% (36/38)**	**>.999**
≥2 Triad components^e^	**75% (27/36)**	**55% (21/38)**	**.075**
All 3 Triad components^f^	**42% (15/36)**	**16% (6/38)**	**.014** *

*Note*: Bolded lines in the table represent the three Triad components, each followed by the respective risk factors.

Abbreviations: BMD, bone mineral density; BMI, body mass index; TT_3_, triiodothyronine.

^a^ Participants with low BMI and/or history of eating disorders and/or low TT_3_.

^b^ Participants who reported delayed menarche and/or history of menstrual irregularity and/or current oligo‐amenorrhea.

^c^ Participants with low BMD and/or who reported history of stress fractures.

^d^ Participants with energy deficiency and/or menstrual disturbances and/or poor bone health.

^e^ Participants with at least two of the three Triad components: energy deficiency, menstrual disturbances, and poor bone health.

^f^ Participants with all three Triad components: energy deficiency, menstrual disturbances, and poor bone health.

* Significant differences between Japanese and American runners.

**FIGURE 4:**
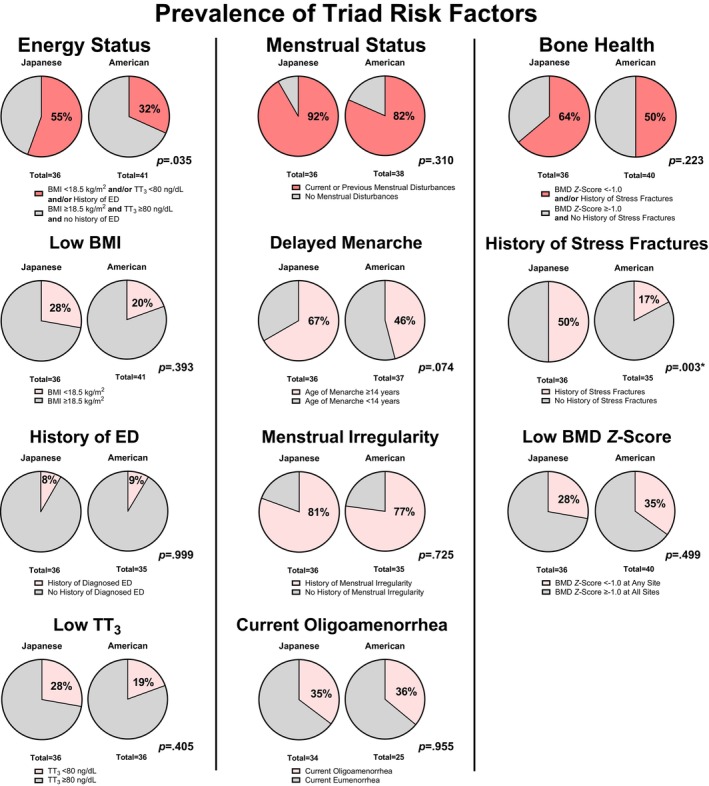
Proportion of Japanese and American female distance runners who experienced each Female Athlete Triad risk factor and Triad component. Panels in the top row depict the three Triad components: Energy status, menstrual status, and bone health. Panels in rows 2–4 depict Triad risk factors of each of the three Triad components. *Statistically significant differences in group proportions for a given Triad risk factor or Triad component (*p* < .05). BMI, body mass index; ED, eating disorders; TT_3_, total triiodothyronine.

Table 4 and Figure 4 depict the prevalence of Triad risk factors and components. A higher proportion of Japanese runners presenting with at least one energy deficiency risk factor (low BMI and/or history of eating disorders and/or low TT_3_) was observed compared to American runners (*p* = .035), but no statistically significant differences were observed in the prevalence of each individual Triad risk factor of energy deficiency (low BMI, history of eating disorders, and low TT_3_). Most runners reported at least one of three menstrual disturbance risk factors (ie, delayed menarche and/or history of menstrual irregularity and/or current oligo‐amenorrhea), with no statistically significant group differences for those variables (*p* = .310). Despite no statistically significant group differences between the proportions of runners with evidence of poor bone health (low BMD and/or history of stress fractures) (*p* = .223), a significantly higher proportion of the Japanese runners reported having experienced stress fractures, compared to the American runners (*p* = .003).

**FIGURE 5:**
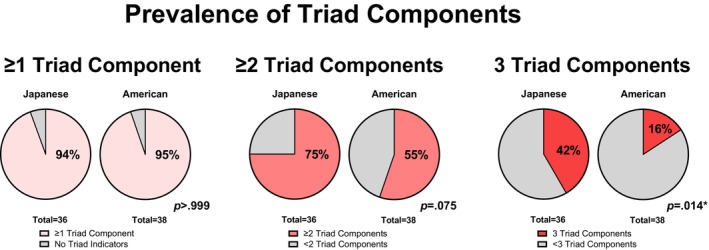
Proportion of Japanese and American female distance runners who experienced Triad risk factors of at least one, at least two, or of all three Female Athlete Triad components: Energy deficiency, menstrual disturbances, and poor bone health. *Statistically significant differences in group proportions (*p* < .05).

Table 4 and Figure 5 depicts the proportion of female runners in this analysis who had experienced Triad risk factors of at least one, at least two, or all three Triad components. Of all runners, 95% experienced at least one Triad risk factor, 65% experienced Triad risk factors of at least two different Triad components, and 28% experienced Triad risk factors of all three Triad components. No statistically significant differences in proportions of Japanese and American runners were observed for those experiencing at least one (*p* > .999) or at least two (*p* = .075) Triad components. However, a higher proportion of Japanese runners experienced all three Triad components compared to American runners (*p* = .014).

## DISCUSSION

To our knowledge, this was the first study to compare all three Triad components between Japanese and American female middle‐ and long‐distance runners. Our findings demonstrated that the prevalence of most Triad risk factors was not significantly different between American and Japanese runners. However, compared to American runners, a higher proportion of Japanese runners had (1) at least one energy deficiency risk factor, that is, low BMI and/or history of eating disorders and/or low TT_3_; (2) reported experiencing stress fractures; and (3) experienced all three Triad components, that is, were positive or had been positive for at least one risk factor for each of the three Triad components. Additionally, Japanese runners had less body fat, lower IGF‐1, and lower BMD than American runners, possibly indicating a higher Triad risk in Japanese runners.

### Energy status

Even though TT_3_ concentrations and BMI were not significantly different between groups, Japanese runners had a higher prevalence of energy deficiency risk factors, defined as low BMI and/or low TT_3_ and/or history of diagnosed eating disorders, despite a higher EI/BW and carbohydrate intake relative to BW. Japanese runners also reported higher exercise volume (min/week) and EEE than American runners, which could suggest that the higher energy expenditure in the Japanese runners was compensated for by a higher EI/BW. However, the higher prevalence of energy deficiency risk factors (low BMI and/or low TT_3_ and/or history of diagnosed eating disorders) and the lower percentage of body fat, FM, and IGF‐1 in the Japanese compared to the American runners suggest that the higher EI/BW in Japanese runners may have been insufficient to account for their substantially higher EEE, exposing that group to an energy deficit. Our findings are similar to the outcomes from the study by Tsukahara et al.,^23^ who showed higher exercise volume, lower percentage of body fat, and a higher tendency for disordered eating behavior, measured from the Eating Attitudes Test in Japanese female elite track and field athletes (sprint and jump events), compared to American collegiate athletes competing in the same events. It is important to note that U.S. Division 1 student‐athletes are regulated by rules that restrict the number of training hours, whereas such restrictions are not applied to student‐athletes in Japan. Furthermore, a greater susceptibility to disordered eating behavior and possibly eating disorders may explain the higher proportion of Japanese than American runners with energy deficiency risk factors in our study. In our study, however, no group differences were observed in the prevalence of diagnosed eating disorders.

Based on previous studies in nonathletic adult populations, cultural differences in the diet of Japanese and Americans might not be the main factor explaining our findings respective to energy and carbohydrate intake. Zhang et al.^21^ reported similar to marginally higher EI in American females aged 20–29 years compared to Japanese peers (no statistical analyses reported). Contrary to our findings, that study showed that carbohydrate intake tended to be higher in American females than Japanese females.^21^ Another study in nonathletes, by Zhou et al.,^22^ showed similar EI between Japanese and American females aged 40–59 years, and a higher proportion of dietary calories from fat sources in Americans compared to Japanese. Further investigations in female athletes from various geographical and cultural backgrounds are warranted.

### Menstrual function

In our study, no statistical differences were observed in current or previous menstrual status between Japanese and American runners. Nevertheless, our findings are concerning, with 92% of Japanese runners and 82% of American runners reporting delayed menarche and/or history of menstrual irregularity and/or current oligo‐amenorrhea, of which 35% and 36%, respectively, reporting current oligo‐amenorrhea. The prevalence of oligo‐amenorrhea was notably higher than the 5.2% observed in a group of 116 collegiate athletes from Japan participating in track and field, swimming, and team sports.^12^ Our findings in American runners, however, were similar to what was reported in another study of 93 American collegiate cross‐country runners that showed a 25.8% prevalence of menstrual irregularities in the past year.^44^ Previous studies demonstrated an association of low L_1_‐L_4_ BMD with current and/or previous amenorrhea^45^, ^46^, ^47^, ^48^, ^49^; therefore, the overall high prevalence (29%) of low L_1_‐L_4_ BMD in our study was possibly associated with the high percentage (86%) of menstrual disturbance risk factors.

### Bone health

Similar to previous comparisons between Asian‐American—from Chinese, Japanese, Philippine, Vietnamese, and Korean origin—and American white populations,^26^ American runners from our study (mostly white) had higher BMD at the lumbar spine, total hip, and femoral neck than the Japanese runners. These outcomes are likely explained by differences in body size between Asian and white populations,^26^ evidenced by differences in height and weight in our study, and similar population‐adjusted L_1_‐L_4_ and femoral neck *Z*‐scores. Even though American runners had lower total hip *Z*‐scores compared to the Japanese runners, the proportion of runners with low total hip Z‐score was not different between groups. The prevalence of low BMD (Z‐score < −1.0) in the American runners from our study was similar to previously reported values for American female collegiate cross‐country runners of 19%^50^ and 40%^44^ at any site (vs. 35% in our study). Moreover, the 30% prevalence of low L_1_‐L_4_ BMD and of 13% at the total hip were similar to a previous study in American female collegiate cross‐country runners that reported ~36% and ~11%, respectively.^51^ The same study also reported a prevalence of low BMD of ~25% at the femoral neck and ~12% for the total body,^51^ which were not notably higher than the 13% and 3% from our study, respectively.

Conversely, in the Japanese runners from our study, the 28% prevalence of low BMD was higher than the 0% prevalence in Japanese collegiate athletes from track and field, swimming, and team sports.^12^ In that study, however, BMD was measured at the heel with an ultrasound, and not a DXA scanner.^12^ Additionally, the 28% prevalence of low L_1_‐L_4_ BMD in our study was higher than the 5% previously reported for a group of Japanese female pubescent gymnasts and track and field athletes.^52^ The discrepancies between our findings in Japanese runners and previously published literature may be explained by differences in age, sport modes, and/or method of BMD assessment. Notably, our study included athletes from only middle‐ and long‐distance running (ie, endurance sports), which likely exposed them to a higher risk of low BMD, as demonstrated previously,^50^ and a high prevalence of menstrual irregularity and oligo‐amenorrhea.

In our study, history of stress fractures was more prevalent in Japanese runners (50%) than in American runners (18%), which might have been due to a combination of the poorer energy status and the lower BMD/smaller bones in the Japanese runners. The prevalence of stress fractures in Japanese females athletes has been reported as 35.3% across multiple collegiate sports, and as 87.5% (7 of 8 athletes) specifically in long‐distance runners,^12^ whereas in adolescents participating in track and field or gymnastics it is reportedly 19%.^52^ In American and Canadian adolescent and collegiate endurance runners, a 17.3% prevalence of bone stress injury (stress reaction or stress fracture) has been reported,^53^ similar to the prevalence of stress fractures reported by our sample of American runners.

### Limitations

Because this study was conducted in two different countries, limitations due to geographic and cultural differences existed, such as the use of two different DXA scanners, different assay platforms for TT_3_ and IGF‐1 measurements, the possibility of undiagnosed medical conditions affecting menstrual function (such as thyroid disorders, endometriosis, or polycystic ovary syndrome), and the different methods of assessing dietary EI and macronutrient intake. Moreover, the self‐report nature of the food records and of the menstrual and medical history are also limitations. Other limitations include the calculation of a part of the BMD *Z*‐scores of American runners, due to the lack of a pediatric database, and the self‐report nature of the health history and dietary and exercise metrics. Notably, even though using different methods of calculating EI and macronutrient intake may limit comparability of the data, it is appropriate to use cultural‐specific parameters, because the foods consumed in the United States may be substantially different than in Japan. Strengths of this study include the use of DXA devices, allowing for precise metrics of body composition and BMD, the use of reliable physiologic measures related to energy deficiency (TT_3_ and IGF‐1), accounting for cultural differences between the American and the Japanese diet, and the inclusion of several Triad risk factors, allowing for a comprehensive analysis of energetic status, menstrual status, and bone health. Notably, this study is one of few publications describing racial/ethnic differences in Triad‐related outcomes between female runners from different countries, and, to our knowledge, is the first to do so comprehensively in American and Japanese female runners.

## CONCLUSIONS

This study was the first to compare multiple Triad risk factors from all three Triad components between Japanese and American female middle‐ and long‐distance runners. We demonstrated that, despite no significant differences in the prevalence of menstrual disturbances, low BMD, low BMI, history of diagnosed eating disorders, and low TT_3_, the Japanese runners had a higher prevalence of energy deficiency risk factors and of stress fractures than American runners, as well as less body fat and higher exercise volume. Our findings suggest that Japanese female runners may be at a higher Triad risk compared to their American peers. Future studies are needed to determine differences between American and Japanese female athletes in clinical and subclinical menstrual disturbances in respect to estrogen and progesterone exposure and the occurrence of ovulation and luteal phase defects. Racial/ethnic differences in Triad outcomes are still understudied and require further investigation not only in runners, but in other endurance and leanness sports, in order to provide information for culture‐specific risk assessment and treatment.

## FUNDING INFORMATION

The Japanese data in this study was supported by a project for Fostering, Survey Research for the Strategic Strengthening of Female Athletes 2019–2020 by the Japan Sports Agency. ACCS received funding from Fulbright Brazil and Coordenação de Aperfeiçoamento de Pessoal de Ensino Superior (CAPES).

## ETHICS STATEMENT

This study was approved by the Pennsylvania State University (#34528) and the Juntendo University (#31–7, July 18th 2019) Institutional Review Boards.

## DISCLOSURE

The authors declare no conflicts of interest.

## PATIENT CONSENT STATEMENT

Participants signed written informed consent forms prior to study participation.

## Data Availability

The data that support the findings of this study are available from the corresponding author upon reasonable request.
